# Multistep surgical management in a patient with recurrent laryngeal papillomatosis: a case report and surgical analysis

**DOI:** 10.25122/jml-2026-0086

**Published:** 2026-09

**Authors:** Dan-Alexandru Popescu, Mihai-Dumitru Tudosie, Simona-Andreea Rujan, Bianca-Petra Taher, Raluca Grigore, Alexandra Maria Marin, Gloria-Simona Berteșteanu, Diana Cosmina Drăgan, Bogdan Popescu, Irina Doinița Popescu, Șerban Vifor Gabriel Berteșteanu

**Affiliations:** 1 Carol Davila University of Medicine and Pharmacy, Bucharest, Romania; 2 ENT Department, Colțea Clinical Hospital, Bucharest, Romania; 3 ENT Department, Emergency Military General Hospital, Bucharest, Romania

**Keywords:** recurrent respiratory papillomatosis, human papillomavirus, laryngeal papillomatosis, CO_2_ laser surgery, human papillomavirus infection, bevacizumab

## Abstract

Recurrent respiratory papillomatosis (RRP) is a benign yet potentially aggressive disease caused by infection with human papillomavirus (HPV), most commonly types 6 and 11. It is characterized by recurrent papillomatous growth within the respiratory tract, predominantly affecting the larynx and often requiring repeated surgical interventions. We present the case of a 29-year-old man with a history of juvenile-onset RRP who developed persistent, progressive dysphonia and exertional dyspnea. Endoscopic evaluation revealed multifocal laryngeal involvement, including lesions of the epiglottis and arytenoid region. The patient underwent endoscopic CO_2_ laser excision with initially favorable outcomes; however, early recurrence necessitated a second, more extensive surgical intervention. The postoperative course was satisfactory, with improved airway patency and vocal function. The patient was evaluated for adjuvant therapy with bevacizumab but was not considered eligible. This case highlights the recurrent nature of RRP, the importance of individualized surgical management, and the role of adjuvant therapies in selected cases. Long-term surveillance remains essential to optimize functional outcomes and enable early detection of recurrence.

## INTRODUCTION

Human papillomavirus (HPV) is currently recognized as an important risk factor in head and neck pathology. Although HPV infection has been predominantly associated with cervical cancer in women, studies published over the last several decades have drawn attention to the role of several HPV types in otorhinolaryngological disorders [1]. Respiratory papillomatosis is a benign condition caused by HPV infection of the epithelial cells of the upper respiratory tract, resulting in proliferative growth that may affect one or more of its segments [2].

Recurrent laryngeal papillomatosis is one of the most common benign neoplasms encountered in head and neck pathology. It is typically classified into two clinical forms: juvenile-onset recurrent respiratory papillomatosis (JoRRP) and adult-onset recurrent respiratory papillomatosis. The HPV types most frequently implicated in both forms are HPV-6 and HPV-11. Furthermore, recent studies indicate three age-related peaks in disease onset: a pediatric peak at approximately 7 years of age, associated with JoRRP, and two adult-onset peaks at approximately 35 and 64 years of age [3–5].

In most cases, recurrent respiratory papillomatosis (RRP) is limited to the larynx; however, it may extend to the trachea, and pulmonary involvement has been reported in approximately 1% of cases, although the underlying pathological mechanism remains unclear. The disease course is variable and may include spontaneous regression, persistence, progressive enlargement, dissemination to other segments of the respiratory tract, or, in rare cases, malignant transformation into squamous cell carcinoma [6–9].

Treatment options have been available for decades, traditionally focusing on surgical removal of papillomas, with strict follow-up protocols to enable early detection of recurrence. More recent literature highlights a shift toward office-based procedures, such as blue laser therapy, along with increased emphasis on functional surgery to preserve voice quality and optimize respiratory function [10].

Additionally, the widespread use of the nonavalent HPV vaccine has been associated with reduced recurrence rates following surgical intervention. In more challenging cases—particularly those involving multiple recurrences or extensive lesions affecting multiple anatomical subsites—adjuvant therapies such as cidofovir or bevacizumab have shown potential benefits [4,11–13].

## MATERIAL AND METHODS

This article is based on a clinical case managed at the ENT Department, Colțea Clinical Hospital, Bucharest, Romania, and describes the patient’s clinical course from admission through follow-up. The total follow-up period was 4 months from the first surgical intervention. All clinical data were collected after obtaining written informed consent from the patient. Clinical photographs were acquired using a Samsung Galaxy S20 smartphone (Samsung Electronics, India).

Regarding the surgical technique, a Lumenis AcuPulse CO_2_ laser (Lumenis, Israel) coupled to a Zeiss OPMI Sensera S7 surgical microscope (Carl Zeiss Meditec AG, Germany) was used. The microscope was configured with a 400 mm focal length and 4.0× magnification. The CO_2_ laser was operated in SuperPulse mode with continuous exposure at a power setting of 10 W, with a spot size of 250 µm, and was delivered through a micromanipulator. The patient was placed in the Boyce–Jackson position and intubated using a flexometallic endotracheal tube (size 7.5; Medtronic).

## RESULTS

A 29-year-old man presented with persistent, progressive dysphonia and mild exertional dyspnea of approximately 3 years’ duration. His medical history was significant for juvenile-onset laryngeal papillomatosis, diagnosed at 15 years of age, for which he had undergone a single surgical excision with cold instruments 4 years before the current presentation.

The history further revealed that his mother had been diagnosed with HPV infection during pregnancy and had received prophylactic treatment, although the specific medication was unknown. The patient also reported chronic keratoconjunctivitis, presumed, on ophthalmological clinical evaluation, to be secondary to HPV exposure during birth. At presentation, his best-corrected visual acuity was logMAR 1.00 in both eyes.

The patient described a noticeable alteration in his voice, particularly affecting his singing voice, which was of special concern given his involvement in a local church choir.

Videoendoscopic examination revealed a small lesion on the laryngeal surface of the epiglottis and a large tumoral growth involving the left arytenoid and extending to the ipsilateral aryepiglottic fold (Figure 1).

**Figure 1:**
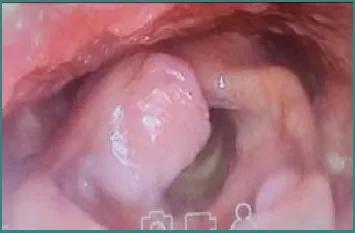
Preoperative videoendoscopic view of the larynx. A small lesion is seen on the laryngeal surface of the epiglottis, with a large tumoral growth involving the left arytenoid and extending to the ipsilateral aryepiglottic fold.

Following a thorough discussion with the patient regarding the risks and benefits of treatment, an endoscopic surgical approach was undertaken. The papillomatous lesions were excised using a CO_2_ laser under microlaryngoscopic visualization. The postoperative course was favorable (Figure 2), and the patient was discharged 2 days later with a referral for voice therapy.

**Figure 2:**
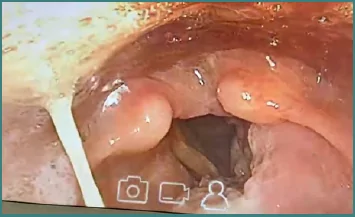
Postoperative videoendoscopic view of the larynx 2 days after the first CO_2_ laser excision

At the first follow-up examination, 2 weeks postoperatively, there were no signs of recurrence. The patient reported minimal voice alteration and expressed a high degree of satisfaction with both his vocal quality and breathing.

However, at the 2-month follow-up after the first surgical intervention, the patient presented with recurrent hoarseness and mild exertional dyspnea. Videofibroscopic examination demonstrated complete recurrence of the previously identified lesions (Figure 3). Given the patient’s age and his desire to continue singing, a second surgical intervention was considered. The increased risk of further recurrence was discussed in detail with the patient.

**Figure 3:**
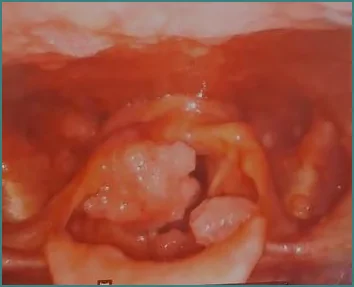
Videofibroscopic view of the larynx at the 2-month follow-up after the first procedure, showing complete recurrence of the previously identified lesions

Because of the extent of the lesions, a more radical surgical approach was adopted, with slightly wider excision margins. The postoperative appearance after the second procedure is shown in Figure 4. Once again, the patient made an excellent recovery and was discharged after 2 days. Histopathological examination showed mild dysplasia after each procedure; HPV genotyping was not performed. Additionally, he was referred to the Infectious Diseases Department for evaluation regarding potential adjuvant therapy with bevacizumab.

**Figure 4:**
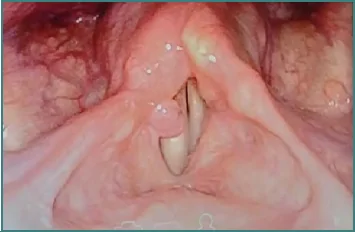
Postoperative view of the larynx 2 days after the second CO_2_ laser excision

At the 2-month follow-up after the second surgery, marked remission of the papillomatous lesions was observed. The patient remains under close surveillance for the detection of recurrence that could affect vocal quality or respiratory function. Following consultation with the Infectious Diseases Department, the patient was not considered eligible for bevacizumab therapy. No further explanation regarding the criteria underlying this decision was provided.

## DISCUSSION

In this specific case, the choice of CO_2_ laser excision was based on the following arguments: high precision with preservation of adjacent structures, minimal intraoperative bleeding, a low risk of synechiae, and faster postoperative healing.

For the second surgery, wider resection margins were chosen because of the extent of the lesions and the rapid recurrence observed 6 weeks after the previous examination. The risk of postoperative scarring or synechiae was discussed with the patient and accepted in view of the expected benefits of the surgery.

The limitations of this case report are the short follow-up and the lack of videofibroscopic documentation from 2 months after the second surgery onwards.

## CONCLUSION

RRP remains a challenging condition owing to its unpredictable clinical course and high recurrence rate. Surgical excision continues to be the mainstay of treatment, with modern approaches emphasizing minimally invasive techniques and the preservation of laryngeal function.

This case illustrates the importance of tailoring therapeutic strategies to disease extent, patient age, and functional requirements, particularly in individuals with professional or recreational voice demands. Although adjuvant therapies such as bevacizumab show promise in reducing recurrence, their use remains limited to selected cases.

Close follow-up is critical for the early detection of recurrence and timely intervention, ultimately improving both respiratory and phonatory outcomes. Further research is needed to refine adjuvant treatment protocols and better understand the factors influencing disease progression and recurrence.

## Data Availability

Additional data are available upon written request, exclusively for educational purposes.
